# Non-coding RNAs regulate autophagy in kidney disease: friend or foe?

**DOI:** 10.1080/15548627.2025.2551683

**Published:** 2025-09-15

**Authors:** Yankun Li, Tongtong Ma, Xinhua Liang, Tingting Jin, Xingqi Zhao, Junmin Huang, Junfeng Hao, Huafeng Liu, Peng Wang

**Affiliations:** a Guangdong Provincial Key Laboratory of Autophagy and Major Chronic Non-communicable Diseases, Key Laboratory of Prevention and Management of Chronic Kidney Diseases of Zhanjiang City, Institute of Nephrology, Affiliated Hospital of Guangdong Medical University, Zhanjiang, Guangdong, China; b Department of Anesthesiology, Affiliated Hospital of Guangdong Medical University, Zhanjiang, Guangdong, China; c Department of Oral and Maxillofacial Surgery, Stomatological Hospital, Southern Medical University, Guangzhou, Guangdong, China; d Division of Orthopaedic Surgery, Department of Surgery, Guangzhou Women and Children’s Medical Center, Guangzhou Medical University, Guangzhou, Guangdong, China

**Keywords:** circRNA, kidney disease, lncRNA, miRNA, precision medicine, therapeutic targets

## Abstract

Macroautophagy/autophagy is a conserved cellular process that degrades misfolded proteins and damaged organelles to regulate cell survival and division. Normal levels of autophagy are observed in healthy kidney cells. In contrast, excessive or insufficient autophagy is observed during kidney disease progression. However, canonical treatments that regulate autophagy using chemical reagents may induce unexpected side effects in other organs. This necessitates the development of therapeutic approaches with fewer adverse effects. Non-coding RNAs, which are highly tissue-specific, regulate autophagy and accurately modulate the expression of related genes. This review presents evidence of the effects of non-coding RNAs on the progression of kidney diseases and their responses to treatment *in vitro*, *in vivo*, and in clinical trials. Our analyses and interpretations of key findings elucidate the pathogenesis of kidney diseases and explore potential new therapeutic approaches.

**Abbreviations:** 3' UTR: 3' untranslated region; 3-MA: 3-methyladenine; ADPKD: autosomal dominant polycystic kidney disease; AKI: acute kidney injury; ccRCC: clear cell RCC; ATG: autophagy related gene; ceRNA: competing endogenous RNA; circRNA: circular RNA; CKD: chronic kidney disease; DKD: diabetic kidney disease; HG: high glucose; IRI: ischemia-reperfusion injury; lncRNA: long non-coding RNA; LPS: lipopolysaccharide; miRNA: microRNA; MTOR: mechanistic target of rapamycin kinase; ncRNA: non-coding RNA; PI3K: phosphoinositide 3-kinase; RCC: renal cell carcinoma; ROS: reactive oxygen species; RTEC: renal tubular epithelial cells; ULK1: unc-51 like autophagy activating kinase 1; UUO: unilateral ureteral obstruction; VHL: von Hippel-Lindau tumor suppressor.

## Introduction

The global prevalence of kidney disease surpasses that of all other non-communicable diseases. The death rate of kidney disease has continuously increased over the past two decades [1]. Furthermore, renal cell carcinoma (RCC) accounts for approximately 3.8% of all newly identified cancers. Clear cell RCC (ccRCC), which primarily stems from renal tubular epithelial cells (RTECs), remains the predominant RCC subtype at approximately 85% [2–4]. Moreover, kidney dysfunction is the seventh leading cause of mortality worldwide, and its prevalence is projected to continuously increase. The progression of renal failure is postponed through kidney replacement therapy, which has substantial economic implications. However, up to 98% of individuals in need of this therapy may lack treatment access in resource-limited settings [5].

Oxidative stress, profibrotic cytokines, urinary proteins, inflammation, and aging play crucial roles in kidney disease [6–8]. Therefore, cellular energy homeostasis is frequently compromised during disease onset and progression. This leads to the initiation of autophagy in renal cells [9–11]. Successful autophagy effectively promotes energy proficiency through ATP production. In addition, it modulates cell and wound repair by removing damaged or redundant organelles and proteins to ameliorate kidney disease [6,12]. However, sustained autophagy activation in injured RTECs may lead to renal fibrosis after acute kidney injury (AKI) [13,14]. Protein kinase inhibitors, such as rapamycin and 3-methyladenine (3-MA), modulate renal autophagy to control kidney disease progression [15,16]. Nevertheless, the regulation of autophagy via chemical treatments that target molecules upstream of this highly conserved cellular recycling process may cause adverse effects in many cells.

The kidney’s high metabolic demand renders it particularly vulnerable to autophagic dysregulation. Emerging evidence implicates non-coding RNAs (ncRNAs) as master regulators of autophagy, fine-tuning disease progression through epigenetic, transcriptional, and post-transcriptional mechanisms. Unraveling these interactions may unlock novel strategies to overcome the limitations of conventional autophagy-targeted therapies.

ncRNAs are a cluster of transcripts that account for > 98% of the genome. They mainly include microRNA (miRNA), long ncRNA (lncRNA), and circular RNA (circRNA). miRNAs are highly conserved among various species. The inhibitory effect of *MIR30A* on BECN1 expression, which leads to decreased autophagy, has been demonstrated in cancer cells [17]. Furthermore, high-throughput sequencing technology has facilitated the identification of lncRNAs and circRNAs. lncRNAs display remarkable spatiotemporal characteristics, especially those characterized by tissue specificity [18,19]. For example, lncRNA *MEG3* activates autophagy and promotes cellular growth in bladder cancer [20]. circRNAs derive stability from their closed circular structure. *circHIPK2* combines autophagy and endoplasmic reticulum (ER) stress interactions to activate astrocyte activation [21]. The regulatory role of ncRNAs in autophagy has been shown in kidney diseases. Therefore, this review outlines the mediatory roles of miRNAs, lncRNAs, and circRNAs in autophagy, which aggravates or mitigates the progression of kidney diseases.

### The definition and diverse mechanisms of ncRNAs

Although incapable of protein-coding, ncRNAs exert considerable control over cellular structures. These important regulators of autophagy help maintain cellular balance. In addition, ncRNAs can be categorized into two primary groups based on their regulatory roles: housekeeping and regulatory [22–24]. Housekeeping ncRNAs are widely expressed in cells. They modulate general cellular functions and play essential roles in cell viability [25,26]. In contrast, regulatory ncRNAs are regarded as the main RNA molecules that modulate gene expression. They regulate gene expression at the epigenetic, transcriptional, and post-transcriptional levels [27,28].

**Figure 1.**
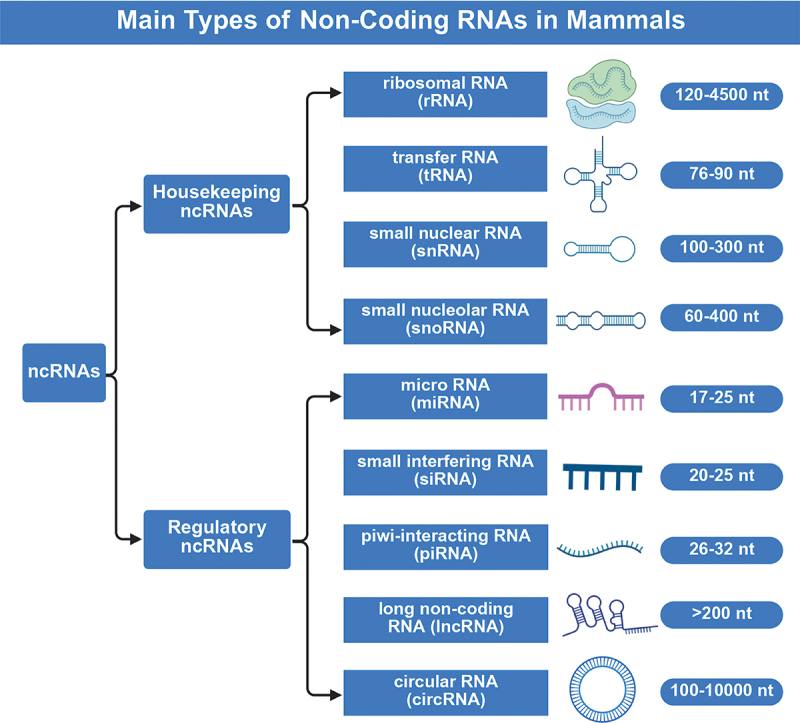
Classification of ncRNAs. Non-coding RNAs can be divided into housekeeping ncRNAs and regulatory ncRNAs. Housekeeping ncRNAs, whose sizes range from 50 to 500 nucleotides (nt), comprise ribosomal RNAs (rRNAs), transfer RNAs (tRNAs), small nuclear RNAs (snRNAs), and small nucleolar RNAs (snoRNAs). Regulatory ncRNAs are categorized by length into small ncRNAs, which are less than 200 nt in length, and long ncRNAs (lncRNAs), which are greater than 200 nt. The primary classes of small ncRNAs include microRNAs (miRNAs), small interfering RNAs (siRNAs), and piwi-interacting RNAs (piRNAs). Additionally, circular RNAs (circRNAs) represent a unique class with covalently closed-loop structures, ranging in sizes from 100 to over 10,000 nt (created with BioRender.com).

Figure 1 illustrates the classification of ncRNAs. In this review, we focus on the following regulatory ncRNAs: miRNAs, lncRNAs, and circRNAs.

### miRNAs

miRNAs comprise 17–25 nucleotides and mainly exert their effects post-transcriptionally [29]. In addition, they are key parts of RNA interference. miRNAs interact with their target mRNAs by forming bases at the 3' untranslated region (3' UTR) [30]. This leads to the degradation or translational suppression of target genes. The miRNA-induced silencing complex interacts with specific mRNAs through miRNA response elements in the 3' UTR. These response elements act as matching sequences to the 2–8 nucleotides at the 5' terminus of miRNA.

### lncRNAs

lncRNAs are transcripts longer than 200 nucleotides. They intricately control gene activity at various stages, including chromatin modification, transcriptional control, and post-transcriptional regulation. The functions of lncRNAs depend on their subcellular localization [31], which can be nuclear or cytoplasmic. lncRNAs regulate the transcription of both cis- and trans-acting genes in the nucleus. Thus, they contribute to chromatin remodeling and post-transcriptional gene regulation. Contrastingly, lncRNAs affect protein localization, regulate mRNA synthesis, stabilize mRNA, and modulate protein-protein interactions in the cytoplasm [32,33].

### circRNAs

circRNAs are formed through back-splicing, a process in which the 5' and 3' ends of the RNA molecule bond covalently to create a stable circular structure [34]. They play diverse regulatory roles in molecular interactions [35]. circRNAs act as molecular sponges. Specifically, they serve as competing endogenous RNAs (ceRNAs) by sequestering miRNAs. This process modulates the availability of miRNAs for target mRNAs, ultimately influencing disease progression. circRNAs can also bind directly to mRNA molecules [36], further regulating their functions. They also interact with proteins [37], including those that bind RNA, which influences their roles in cellular processes. Moreover, circRNAs interact with transcription factors [38], which consequently affects the regulation of specific genes. Even circRNAs can encode functional proteins [39].

## Autophagy

### Classification

Autophagy is a cellular process that degrades and recycles damaged or unnecessary cellular components. It is categorized based on how the intracellular substrate is delivered to the lysosomal cavity: macroautophagy, microautophagy, and chaperone-mediated autophagy (CMA) [40]. Microautophagy is a rapid form of autophagy that does not form autophagosomes. Instead, it occurs through direct degradation by lysosomes or late endosomes. During this process, the lysosomal membrane folds inward to surround cellular components like parts of the mitochondrion or ER [41]. CMA is highly selective; it recognizes and binds proteins with specific sequences through molecular chaperones [42]. These proteins are then transported into lysosomes by transport proteins on the lysosomal membrane for degradation [43].

Macroautophagy is categorized as either nonselective or selective, based on the specificity of the degraded substrate. Microautophagy can also be either nonselective or selective. Nonselective autophagy entails the random uptake of cytoplasmic material for degradation, while selective autophagy targets specific cellular components for degradation. Selective autophagy specifically targets and degrades various substrates, including mitochondria, peroxisomes, ribosomes, ER, lysosomes, nucleus, proteasomes, and lipid droplets [44,45].

The ncRNA regulation of microautophagy and CMA, especially in kidney disease, remains largely understudied. Therefore, this review primarily focuses on studies related to macroautophagy. In addition, this review mainly focuses on nonselective autophagy because it is more widely studied than selective autophagy.

### Process

Macroautophagy, commonly known as autophagy, is the most extensively researched type and can be broadly categorized into the following stages:

1. Initiation: when the cell undergoes nutrient deficiency or stress, it activates a series of signaling pathways, such as inhibition of MTOR (mechanistic target of rapamycin kinase) and AMP-activated protein kinase (AMPK). These pathways activate ATG (autophagy autophagy related gene) proteins, including the ULK1 (unc-51 like autophagy activating kinase 1) complex, which initiates the autophagy process.
2. Nucleation and vesicle formation: regulated by the initiation signal, the cell membrane structure begins to form a double-membrane sequestering compartment, namely a phagophore. This process usually begins in the ER, and the phagophore gradually expands, enveloping the surrounding cytoplasm and organelles.
3. Expansion and maturation: phagophores continue to grow, ultimately sealing and forming double-membrane vesicle termed an auotophagosome. This compartment may fuse with other membrane structures, such as endosomes or lysosomes, eventually forming an autolysosome.
4. Degradation and recycling: mature autophagosomes fuse with lysosomes to form autolysosomes. After this fusion, enzymes within lysosomes degrade the autophagosomal membrane and cargo into basic biomolecules such as amino acids and fatty acids. These molecules are subsequently released back into the cytoplasm, where they can be reused by the cells and participate in new biosynthetic processes, completing the cycle of autophagy [46–49].

### Signaling pathways on autophagy regulation

Autophagy is controlled by a sophisticated network of intracellular signals that react to nutrient availability. This network can either stimulate or inhibit autophagy. This action helps cells adapt to stress or counteract its effects. Key complexes involved in the regulation include MTOR, class III phosphatidylinositol3-kinase (PtdIns3K), ATG12–ATG5, and MAP1LC3/LC3 (microtubule associated protein 1 light chain 3). The MTOR kinase is a vital regulator of autophagy. When activated by AKT and MAPK (mitogen-activated protein kinase) signaling, MTOR inhibits autophagy. Conversely, its negative regulation by AMPK and TP53/p53 signaling promotes autophagy [50]. The serine/threonine kinases ULK1, ULK2, and ULK3 act downstream of the MTOR complex and form a large complex with the mammalian homolog of ATG13 and the scaffold protein RB1CC1/FIP200 [51]. The PtdIns3K complex is essential for autophagy induction and includes PIK3C3/VPS34, BECN1 (a mammalian homolog of yeast Vps30/Atg6), PIK3R4/VPS15/p150, NRBF2 and either ATG14 or UVRAG. ATG12 conjugates to ATG5 through a ubiquitin-like reaction involving ATG7 and ATG10, and then forms a large complex with ATG16. LC3 is cleaved by ATG4 to produce cytosolic LC3-I, which conjugates to phosphatidylethanolamine (PE) through a ubiquitin-like reaction involving ATG7 and ATG3, forming lipidated LC3-II that attaches to phagophore membranes [52]. High MTOR activity inhibits autophagy under nutrient-rich conditions by phosphorylating ULK1 at Ser757, which disrupts the ULK1-AMPK interaction. In contrast, during starvation, AMPK directly activates ULK1 through phosphorylation at Ser317 and Ser777, promoting autophagy [53]. Autophagy is also induced by reactive oxygen species (ROS), ER stress, hypoxia, DNA damage, and immune signaling [54]. Activated ULK1 phosphorylates BECN1 on S14, enhancing the activity of the ATG14-containing PIK3C3/VPS34 complex and promoting phagophore formation. Subsequently, *ATG* genes control autophagosome formation through the ATG12–ATG5 and LC3-II complexes [55].

Other pathways that regulate autophagy include BCL2, which inhibits autophagy by suppressing BECN1 activation [56]. The RAS-RAF-MAP2K1/MEK-MAPK/ERK pathway inhibits autophagy by activating the mechanistic target of rapamycin kinase complex 1 (MTORC1) or promotes LC3-II expression and autophagy activation [57,58]. MAPK/p38 inhibits autophagy by suppressing ULK1 activation or promotes it by upregulating BECN1 and ATG7 expression [59,60]. The WNT-CTNNB1/β-catenin pathway indirectly inhibits autophagy by suppressing LC3-II and BECN1 expression [61]. TP53 promotes autophagy by enhancing the transcription and synthesis of autophagy-related proteins [62]. PTEN (phosphatase and tensin homolog) inhibits the class I phosphoinositide 3-kinase (PI3K)-AKT-MTORC1 pathway, promoting autophagy by inhibiting AKT-MTORC1 phosphorylation [63]. SIRT1 (sirtuin 1), and other sirtuins (NAD^+^-dependent class II histone deacetylases), promote autophagy by deacetylating ATG5, ATG7, and mammalian Atg8-family homologs (ATG8s) [64].

**Figure 2.**
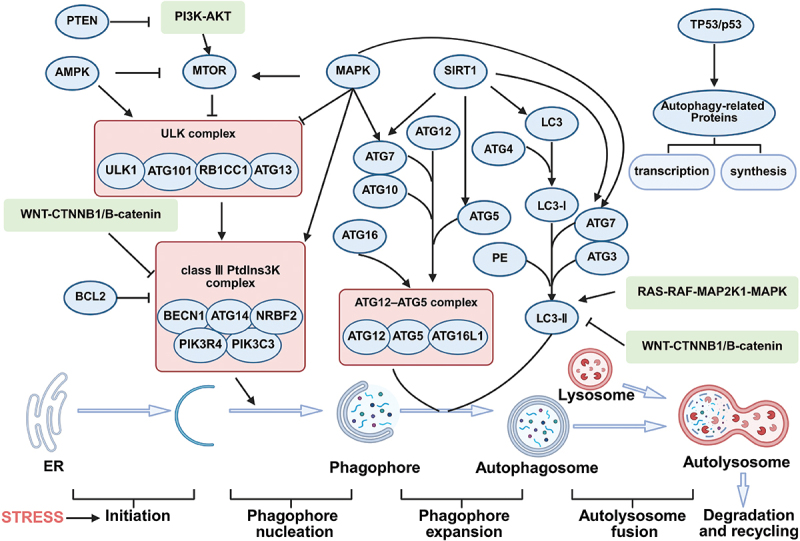
Autophagy process and signaling pathway on autophagy regulation. Autophagy can be roughly divided into several stages: 1) initiation, 2) nucleation and vesicle formation, 3) expansion and maturation, and 4) degradation and recycling. Signaling pathways such as those involving AMPK, PI3K-AKT, WNT-CTNNB1/β-catenin and MAPK regulate autophagy by modulating the MTOR complex, the class III phosphatidylinositol 3-kinase (PtdIns3k) complex, the ATG12–ATG5-ATG16L1 complex, and the LC3-II (ATG8-II) complex (created with BioRender.com).

Figure 2 depicts the process of autophagy and the pathways by which it is regulated.

### Autophagy in kidney disease

#### Autophagy in RCC

The dual role of autophagy in cancer is widely acknowledged and has significant implications for understanding tumor biology. As RCC progresses, the switch between different isoforms of hypoxia-inducible factors/HIFs and changes in metabolism work together to define autophagy’s dual role [65–68]. In early-stage RCC, mild hypoxic conditions enable the autophagy system to maintain cellular balance by efficiently clearing damaged organelles, especially dysfunctional mitochondria. This process also prevents oxidative stress-induced genomic instability, resulting in significant tumor-suppressive effects [69]. During this phase, HIF1A/HIF-1α, which remains stably expressed, enhances tumor-suppressive metabolic reprogramming by activating BNIP3-dependent selective mitophagy, which in turn inhibits glycolysis [70]. As the tumor progresses to late stages, chronic hypoxia causes a gradual transition from HIF1A to EPAS1/HIF-2α dominance. This shift fundamentally alters the metabolic network, promoting aggressive tumor growth [71]. At this stage, autophagy transforms functionally. It both supports tumor energy by recycling nutrients like glutamine and suppresses anti-tumor immune responses by increasing immune checkpoint molecules such as CD274/PD-L1 [72,73]. In advanced RCC, the upregulation of ITPR1 expression facilitates tumor immune evasion by inducing protective autophagy, which particularly enables resistance to natural killer cell-mediated cytotoxicity [74,75]. Under persistent hypoxia-inducible factor activation, impaired autophagic flux causes abnormal accumulation of SQSTM1/p62. This accumulation constitutively activates the NFE2L2/NRF2 signaling pathway. This significantly enhances the tumor’s antioxidant defense capacity and directly contributes to clinical chemoresistance [69,76].

#### Autophagy in AKI

AKI is characterized by the rapid loss of kidney function due to ischemic or nephrotoxic damage. Ischemia-reperfusion injury (IRI), sepsis, and nephrotoxic drugs, such as cisplatin, induce intracellular stress in RTECs. This includes hypoxia, ER stress, and oxidative stress, which trigger autophagy. Autophagy generally mediates the clearance of protein aggregates and damaged organelles to maintain cellular homeostasis and prevent renal cell damage [50,51].

IRI, an inflammatory process triggered by the temporary reduction or cessation of organ blood flow and the subsequent restoration of blood flow, is a common cause of AKI. Elevated expression of autophagy-related proteins, such as BECN1 and LC3, and increased autophagosome formation have been observed in rat and mouse renal IRI models [77,78]. The kidneys are more sensitive to renal IRI than wild-type mice in proximal tubules with selective *atg5* or *atg7* deletion, exhibiting more significant renal dysfunction and tubular cell apoptosis [52]. Similar phenomena have been observed in mice deficient in *Pink1* (PTEN induced putative kinase 1) or *Prkn* (parkin RBR E3 ubiquitin protein ligase) and in *pink1 prkn* double-knockout mice, which are susceptible to ischemic AKI because the PINK1- and PRKN-dependent mitophagy is inhibited [79]. Moreover, autophagy inhibitors, such as chloroquine and 3-MA, can exacerbate renal IRI and worsen renal tubular apoptosis [80,81].

Sepsis, which is characterized by systemic inflammation, is another common cause of AKI. Lipopolysaccharide (LPS), the most common sepsis-associated AKI (SA-AKI) drug, can induce autophagy in RTECs both *in vitro* and *in vivo* [82,83]. Similar to ischemic AKI, increased expression of autophagy-related proteins and increased autophagosome formation have also been observed in AKI caused by LPS-induced sepsis [84]. Moreover, conditional *atg7* knockout in renal proximal tubule (*atg7*-ptKO) mice were more susceptible to LPS-induced AKI than wild-type mice [82,83]. Chloroquine inhibits autophagy and exacerbates LPS-induced AKI [83]. Chloroquine increases kidney damage in mice that also present with the cecal ligation and puncture model of polymicrobial sepsis [85]. However, rapamycin induces autophagy and improves renal function in a cecal ligation and puncture mouse model [86].

Nephrotoxic drugs such as cisplatin are also common causes of AKI. Increased autophagosome formation has been observed in cisplatin-induced AKI [87]. Conditional *atg5* knockout in renal proximal tubule (*atg5*-ptKO) mice resulted in increased sensitivity to cisplatin nephrotoxicity, along with more severe cell apoptosis, ROS production, and DNA damage [50,53]. In addition, cisplatin-induced apoptosis in renal tubular cells increased after the administration of the autophagy inhibitor 3-MA [87].

#### Autophagy in chronic kidney disease

Chronic kidney disease (CKD) is characterized by progressive loss of renal function and the development of renal fibrosis. It is a highly prevalent irreversible and progressive disease [88,89]. In CKD subtypes, such as diabetic kidney disease (DKD), obstructive nephropathy, and autosomal dominant polycystic kidney disease (ADPKD), autophagy may be induced to protect RTECs and podocytes, whereas impaired autophagy causes progressive CKD [90–93].

DKD is a serious complication of diabetes and a major cause of CKD and end-stage renal disease [94]. In DKD, chronic hyperglycemia and the associated advanced glycation end products lead to dysregulated autophagy in podocytes and TECs, consequently promoting DKD progression. Podocyte-specific *atg5* knockout mice exhibited more pronounced mesangial expansion and glomerulosclerosis following streptozotocin (STZ)-induced DKD than wild-type mice [48]. In addition, proximal TECs exposed to high glucose (HG) and tubules from diabetic mice showed increased expression of inositol oxygenase, mitochondrial fragmentation, and decreased autophagy. The inhibition of inositol oxygenase can reactivate mitochondrial autophagy and improve its integrity [49].

Unilateral ureteral obstruction (UUO) is the most common animal model of renal fibrosis. Autophagy also prevents renal fibrosis in UUO-induced CKD models. For instance, *atg5*-ptKO promotes cell cycle arrest in the G2/M phase and exacerbates renal tubulointerstitial fibrosis after UUO in mice [56]. Furthermore, the conditional knockout of *Atg7* in distal RTECs exacerbates renal tubulointerstitial fibrosis and epithelial-stromal transition-like phenotypic changes in UUO mice [95]. However, some studies have reported contradictory findings. For example, the reduction of renal fibrosis has been reported in UUO mice treated with chloroquine to inhibit autophagy or selective knockout of *Atg7* in the proximal tubules [57].

ADPKD, which is caused by PKD1 or PKD2 mutations, is a common inherited human disease that can lead to renal failure. In the ADPKD mouse model, elevated expression of autophagy-related proteins and increased autophagosome formation were observed in cystic renal tubular cells [96]. Furthermore, rapamycin inhibited cyst expansion in an ADPKD mouse model [97]. In the ADPKD zebrafish model, *atg5*-KO inhibits autophagy to promote cyst formation, whereas the use of BECN1 peptide can activate autophagy to improve cyst formation [98].

## Regulation of autophagy in RCC by ncRNAs

Autophagy is a double-edged sword in cancer. It acts as both a tumor suppressor to prevent tumorigenesis and as a pro-survival factor that enables cancer cells to survive metabolic stress and chemotherapeutic agents. Aberrant autophagy regulated by ncRNAs is potentially involved in the development of RCC.

### Regulation of autophagy in RCC by miRNAs

#### Inhibition of autophagy in RCC by miRNAs

miRNAs can suppress autophagy in RCC, thereby reducing its pathogenic processes. Most ccRCC cases involve the loss of VHL, which is a tumor suppressor gene. For example, a decrease in *MIR204* expression was observed in 128 human ccRCC tissues, contrary to 114 healthy kidney samples. *MIR204* binds to the 3' UTR of *LC3B*, which reduces LC3B expression in VHL(-) RCC cells. During the developmental phase of autophagy, it distinctly suppressed macroautophagy triggered by starvation in VHL(-) RCC cells, as opposed to VHL(+) RCC cells. The inhibition of macroautophagy by exogenous *MIR204* causes RCC cells to become synthetically lethal and succumb to necrosis-induced cell death due to starvation [99]. Additionally, VHL directly binds to the 3' UTR of *TRPM3/Melastatin 3* (transient receptor potential cation channel subfamily M member 3), hindering its expression via *MIR204*. Alternatively, it can indirectly suppress TRPM3 by targeting *CAV1* (caveolin 1), another target of *MIR204*. The inhibition of TRPM3 leads to lower intracellular concentrations of calcium, which suppresses ULK1 phosphorylation and autophagy. This ultimately prevents the growth of tumors in orthotopic xenografts [100]. The exogenous expression of *MIR30A* in 786-O or A489 cells inhibited BECN1 expression and enhanced sorafenib-induced cytotoxicity, resulting in the apoptosis of numerous RCC cells [101]. Additionally, *HMOX1/HO-1* (heme oxygenase 1), an anti-apoptotic gene, plays a role in regulating autophagy in cancer [102]. *MIR200C* targets *HMOX1* to induce apoptosis and autophagy, suppressing ccRCC proliferation and improving drug sensitivity to sorafenib/imatinib [103].

#### Promotion of autophagy in RCC by miRNAs

Conversely, miRNAs may enhance RCC progression by promoting autophagy. *MIR501-5p* exhibited increased expression in KJ29, Caki-1, and Caki-2 renal cancer cell lines compared to that in normal epithelial kidney 4/5 and human embryonic kidney cell line 293 (HEK293) cells. *MIR501-5p* binds to the 3' UTR of *MCU* (mitochondrial calcium uniporter) mRNA, leading to a decreased production of MCU protein. Mitochondrial Ca^2+^ levels were notably diminished after ATP stimulation. This triggers AMPK, leading to the initiation of autophagy independent of MTOR. Additionally, this triggers the breakdown of the tumor-inhibiting protein TP53. This further emphasizes the complex functions of ncRNAs in the regulation of autophagy during RCC progression [104].

### Regulation of autophagy in RCC by lncRNAs

#### Inhibition of autophagy in RCC by lncRNAs

lncRNA *HOTTIP* enhances the phosphorylation of PI3K and AKT while simultaneously diminishing ATG13 expression in RCC patient tissues. This results in the suppression of autophagy, thereby promoting the growth, migration, and penetration of RCC cells [105]. Moreover, lncRNA *LBX2-AS1* promotes the proliferation and migration of ccRCC by inhibiting mitophagy [106].

#### Promotion of autophagy in RCC by lncRNAs

In addition to inhibiting autophagy, lncRNAs enhance RCC progression by promoting autophagy. lncRNA *IGFL2-AS1* increases tumor protein *TP53INP2* (tumor protein p53 inducible nuclear protein 2) mRNA levels in sunitinib-resistant RCC cells by competitively binding to HNRNPC (heterogeneous nuclear ribonucleoprotein C). This protein post-transcriptionally reduces TP53INP2 expression through alternative splicing. The upregulation of TP53INP2 expression leads to increased autophagy, thereby conferring sunitinib resistance in RCC cells [107].

### Regulation of autophagy in RCC by ceRNA-miRNA axes

The regulatory network involving ceRNAs, such as lncRNAs and circRNAs, depends on their ability to act like sponges that bind to and inhibit miRNAs. Such interactions are pivotal in modulating various cellular processes and biological functions.

#### Inhibition of autophagy in RCC by ceRNA-lncRNA axes

lncRNA *SBF2-AS1* acts as a ceRNA that targets *MIR338-3p*, upregulating the expression of ETS1 (ETS proto-oncogene 1, transcription factor). This suppresses ccRCC cell death and autophagy, consequently facilitating their proliferation, migration, and infiltration [108].

#### Promotion of autophagy in RCC by ceRNA-miRNA axes

Increased levels of lncRNA *KIF9-AS1* in RCC cells promote cell survival and reduce cell death during sorafenib treatment. This phenomenon results from increased ATG9A activity and decreased SQSTM1/p62 levels. The diminished SQSTM1/p62 levels are attributed to the targeting of *MIR497-5p* by lncRNA *KIF9-AS1*, which facilitates RCC drug resistance through chemical processes [109]. Additionally, circRNA microarray analysis revealed increased *circ0054537* levels in RCC tissues [110]. Furthermore, *circ0054537* was identified as a ceRNA for *MIR640*, which binds to the 3' UTR of *NPTX2* (neuronal pentraxin 2) mRNA. Eliminating *circ0054537 in vitro* effectively disrupts cellular movement, penetration, autophagy, and glycolysis and slows tumor development in living organisms [111]. Moreover, extensive sequencing revealed higher expression levels of *circ0035483* in RCC. An inverse relationship was observed between this circRNA and *MIR335*. *circ0035483* binds to *MIR335* in sorafenib-treated RCC cells, enabling its targeting of the 3' UTR of *CCNB1* (cyclin B1) mRNA. Thus, suppression of CCNB1 expression triggers autophagy, curbs apoptosis, and increases sorafenib resistance. These processes have a notable effect on the results of chemotherapy in RCC [112].

**Figure 3.**
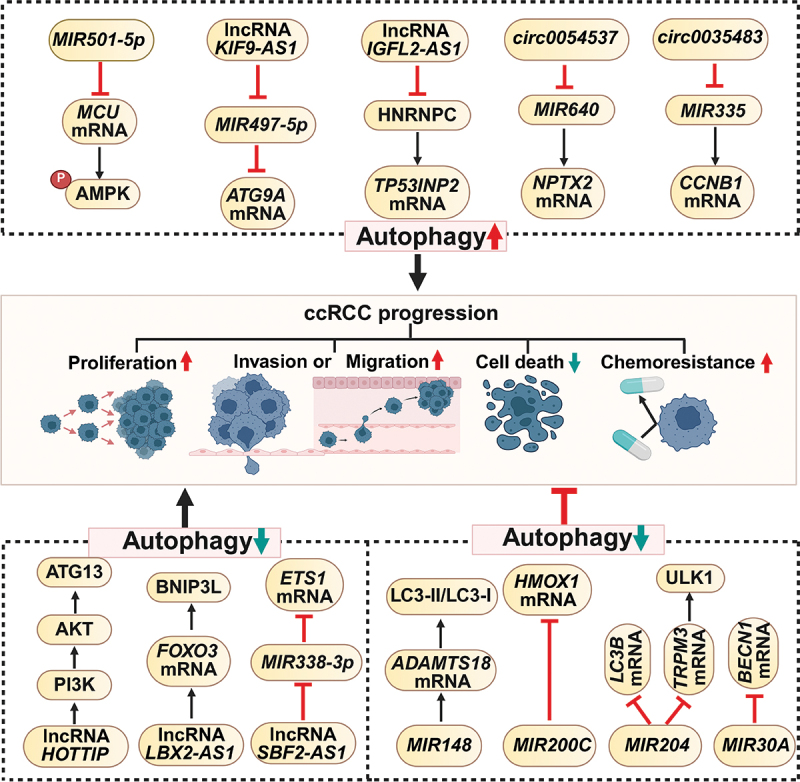
Regulation of ccRCC progression by autophagy-modulating ncRNAs. Certain ccRCC-associated ncRNAs drive tumor progression by enhancing autophagy. For instance, *MIR501-5p* suppresses MCU expression, reducing mitochondrial calcium uptake and subsequently activating AMPK to initiate autophagy. Similarly, *KIF9-AS1* counteracts *MIR497-5p*, upregulating ATG9A and conferring sorafenib resistance in RCC cells. *IGFL2-AS1* elevates mRNA levels of *TP53INP2* by blocking HNRNPC-mediated alternative splicing, leading to sunitinib resistance. Moreover, *circ0054537* and *circ0035483* facilitate ccRCC progression in an autophagy-dependent manner by acting as miRNA sponges, upregulating NPTX2 and CCNB1, respectively. Conversely, some ncRNAs suppress autophagy while promoting tumorigenesis: *HOTTIP* inhibits autophagy and promotes ccRCC progression through the PI3K-AKT-ATG13 axis. *LBX2-AS1* exerts similar effects via the FOXO3-BNIP3L pathway. Additionally, *SBF2-AS1* inhibits autophagy by sequestering *MIR338-3p*, leading to ETS1 upregulation and subsequent ccRCC progression. Notably, several miRNAs (*MIR148*, *MIR200C*, *MIR204*, *MIR30A*) act as tumor suppressors by inhibiting autophagy through distinct pathways: *MIR148* acts via the ADAMTS18-LC3 axis, *MIR200C* inhibits HMOX1/HO-1, *MIR204* targets the TRPM3-ULK1 axis, and *MIR30A* downregulates BECN1 (created with BioRender.com).

The autophagy-regulated ncRNAs and their targets in RCC are concluded in Figure 3 and Table 1.

**Table 1.** Non-coding RNAs regulate autophagy in renal cell Carcinoma (RCC).

ncRNAs	Expression in RCC/non-RCC tissue	Animal/human (numbers of clinical samples)	Cell line	Potential target (Direct or Indirect*)	Gain- or loss- of- function studies	Autophagy modulation	Influence on RCC pathogenesis	Ref.
*MIR148*	Down	Human	Human RCC cell lines (A498, 786-O)	*ADAMTS18* (Indirect)	+/+	Inhibition	Suppression (Proliferation↓)	[113]
*MIR200C*	Down	Human	Human RCC cell lines (SN12C, ACHN, 786-O, Caki-1)	*HMOX1* (Direct)	+/+	Inhibition	Suppression (Proliferation↓, Drug resistance↓, Apoptosis↑)	[103]
*MIR204*	Down	Mouse and human; 128 human RCC with VHL status and 114 cases matched normal kidney specimen	Human RCC cell lines (786-O, A498, Caki-1)	*LC3B* (Direct)	+/+	Inhibition	Suppression (Necrotic cell death↑)	[99]
	Down	Mouse and human; 66 human RCC tumors with VHL-inactive and 24 human RCC tumors with wild type VHL	Human RCC cell lines (786-O, A498, Caki-1)	*TRPM3* (Direct), *CAV1* (Direct)	+/+	Inhibition	Suppression (Proliferation↓)	[100]
*MIR30A*	Down	Human; 10 fresh human RCC tissues of different-grades (AJCC stages 1–4) and surrounding normal tissues	Human RCC cell lines (786-O, A498, SK-RC-44)	*BECN1* (Indirect)	+/+	Inhibition	Suppression (Drug resistance↓)	[101]
*MIR501-5p*	Up	Human	Non papillary kidney cancer KJ29 cells; RCC cell lines (Caki-1)	*MCU* (Direct)	+/+	Promotion	Enhancement (Proliferation↑, Migration↑)	[104]
lncRNA *HOTTIP*	Up	Mouse and human; 42 paired human RCC and adjacent normal samples	Human RCC cell lines (786-O, A498, ACHN, OSRC-2)	PI3K-AKT-ATG13 (Indirect)	+/+	Inhibition	Enhancement (Proliferation↑, Migration↑, Invasion↑)	[105]
lncRNA *IGFL2-AS1*	Up	Human; 72 patients with RCC and all patients underwent nephrectomy before sunitinib therapy	Human RCC cell lines (786-O, 769-P, ACHN)	HNRNPC (Direct)	+/+	Promotion	Enhancement (Drug resistance↑)	[107]
lncRNA *KIF9-AS1*	–	Human	Human RCC cell lines (Caki, 786-O)	*MIR497-5p* (Direct)	+/+	Promotion	Enhancement (Drug resistance↑)	[109]
LncRNA *LBX2-AS1*	Up	Mouse and human; 96 paired RCC tissues and adjacent non-tumor tissues	Human RCC cell lines (769-P, Caki-1)	*FOXO3* (Indirect)	-/+	Inhibition	Enhancement (Proliferation↑, Migration↑)	[106]
lncRNA *SBF2-AS1*	Up	Mouse and human; cancer tissues or adjacent tissues from 46 patients with RCC	Human RCC cell lines (Caki-1, UT14, UT16, UT33a, 786-O); Human renal proximal tubular cell lines (HK-2)	*MIR338-3p* (Direct)	+/+	Inhibition	Enhancement (Proliferation↑, Migration↑, Invasion↑, Apoptosis↓)	[108]
*circ0035483*	Up	Human; 3 kidney cancer specimens and 3 paired contra lateral normal specimens	Human RCC cell lines (TK10, UO31)	*MIR335* (Direct)	+/+	promotion	Enhancement (Drug resistance↑)	[112]
*circ0054537*	Up	Mouse and human; RCC specimens were surgically obtained from 39 patients diagnosed with RCC	Human RCC cell lines (786-O, A498); Human renal proximal tubular cell lines (HK-2)	*MIR640* (Direct)	+/+	promotion	Enhancement (Proliferation↑, Migration↑, Invasion↑, Apoptosis↓)	[111]

*Direct: interactions validated by luciferase reporter assays, RNA immunoprecipitation assays, or RNA affinity-isolation assays. Indirect: expression correlations only.

## Regulation of autophagy in AKI by ncRNAs

Autophagy generally plays a protective role in AKI, despite some exceptions. ncRNAs-regulated autophagy dysfunction is potentially linked to AKI advancement.

### Regulation of autophagy in AKI by miRNAs

#### Inhibition of autophagy in AKI by miRNAs

miRNAs can promote the progression of AKI by inhibiting autophagy. For example, *MIR21* regulates RAB11A expression at the post-transcriptional level without affecting its mRNA level but inhibits its protein level in rat kidneys subjected to I/R or I/R-treated NRK-52E cells. This suppression results in inhibited autophagy in I/R-induced AKI in rat kidneys, reduced cell viability in NRK-52E cells, and increased cell apoptosis [114]. Furthermore, *MIR20A-5p* suppresses autophagy by downregulating ATG16L1 expression, a factor crucial for cell survival during ischemic kidney injury [115]. In addition, *MIR30B* can further aggravate apoptosis and inflammation, as well as impair autophagy in LPS-induced HK2 cells. This is achieved by loading this miRNA into ion-crosslinked polysaccharide nanoparticles [116]. Moreover, *MIR34A* binds to the 3' UTR of *ATG4B*, leading to a post-transcriptional suppression of ATG4B expression. This consequently hinders autophagic activity in I/R-treated RTECs and results in cellular damage [117]. Furthermore, *MIR30E-5p* inhibits autophagy by diminishing the expression of BECN1 and fostered CASP3 (caspase 3) production, which triggers apoptosis in HK2 cells subjected to urografin exposure [118].

miRNAs may also alleviate AKI progression by inhibiting autophagy. An increase in LC3-II/I and BAX levels, along with a reduction in BCL2 expression, was observed in NRK-52E cells challenged with iodixanol. Increasing *MIR1-3p* levels may inhibit autophagy and subsequent apoptosis in NRK-52E cells by targeting *ATG13* and activating the AKT signaling pathway. This action helps mitigate kidney damage in rats with contrast-induced AKI [119]. Moreover, *MIR526B* can bind to the 3' UTR within *ATG7* mRNA in both AKI mouse kidney tissues and LPS-activated HK2 cells. This leads to decreased post-transcriptional expression and inhibits autophagy, consequently mitigating SA-AKI damage caused by excessive autophagy [120]. *MIR92A* suppresses MAP2K4/MEK4-MAPK8/JNK1-related autophagy, mitigating kidney IRI [121].

#### Promotion of autophagy in AKI by miRNAs

miRNAs can delay AKI progression by promoting autophagy. For instance, *MIR590-3p* binds to the 3' UTR of *TRAF6* (TNF receptor associated factor 6) mRNA, which suppresses TRAF6 expression. This leads to increased expression of LC3-I and BECN1, decreased SQSTM1/p62 expression, enhanced autophagy, and improved defense against kidney I/R damage [122]. Additionally, *MIR506-3p* may inhibit PIK3CA (phosphatidylinositol-4,5-bisphosphate 3-kinase catalytic subunit alpha) expression by binding to the 3' UTR of *PIK3CA* mRNA. Furthermore, it may promote autophagy to reverse renal tissue damage, mitochondrial structural alterations, and cell demise in mouse RTECs affected by SA-AKI [123]. *MIR365-3p* targets *Rheb* (ras homolog enriched in brain), promoting autophagy in RTECs and reducing apoptosis induced by contrast media [124]. Moreover, *MIR145* from human umbilical cord-derived mesenchymal stem cells promotes autophagy in HK-2 cells by inhibiting the PI3K-AKT-MTOR signaling pathway [125].

### Regulation of autophagy in AKI by lncRNAs

Like miRNAs, lncRNAs can worsen AKI by inhibiting autophagy. The lncRNA *EGOT* specifically decreases the expression of ATG7 and ATG16L1, thereby hindering the development of autophagosomes in H/R-treated HK2 cells [126].

### Regulation of autophagy in AKI by circRNAs

Despite limited research, circRNAs play a significant role in regulating autophagy and the progression of AKI. Aberrant expression of circRNAs significantly impacts AKI by regulating autophagy. Contrary to typical circRNAs that act as miRNA sponges, *circZNF609* was highly expressed in the urine of 119 AKI patients and in the renal tissue of rats following I/R treatment. In addition, this circRNA produces a functioning protein with 250 amino acids (aa), designated as ZNF609-250aa. ZNF609-250aa triggers the AKT3-MTOR signaling pathway, disrupting autophagy in HK2 cells, which promotes apoptosis and hinders proliferation [127].

### Regulation of autophagy in AKI by the ceRNA-miRNA axes

#### Inhibition of autophagy in AKI by the ceRNA-miRNA axes

lncRNA *SNHG14* decreases the levels of *MIR495-3p* and increases the expression of HIPK1 (homeodomain interacting protein kinase 1). This leads to increased expression of BAX, cleaved CASP3 (caspase 3), and SQSTM1/p62. In contrast, lncRNA *SNHG14* decreases the expression of BCL2 and LC3-II in LPS-treated HK2 cells [128].

#### Promotion of autophagy in AKI by the ceRNA-miRNA axes

As a ceRNA, lncRNA *HCG18* binds to *MIR16-5p*, promoting BCL2 expression, which facilitates cellular autophagy and suppresses apoptosis in IRI-induced AKI [129]. The ceRNA-miRNA axis may promote AKI progression by enhancing autophagy. lncRNA *TUG1*, a ceRNA, interacts with *MIR29A* to enhance the expression of its target gene *PTEN*. This process promotes autophagy and fosters apoptosis and inflammation of kidney tubular cells after H/R treatment [130]. Moreover, lncRNA *NKILA* enhances CLDN2 (claudin 2) expression by acting as a ceRNA for *MIR140-5p*, which promotes autophagy, apoptosis, and inflammatory responses in LPS-treated HK2 cells [131]. Furthermore, the lncRNA *MEG3-MIR145-5p*-RTKN-WNT-CTTNB1/β-catenin-MYC/c-MYC positive feedback loop aggravates renal IRI by activating mitophagy and apoptosis [132].

**Figure 4.**
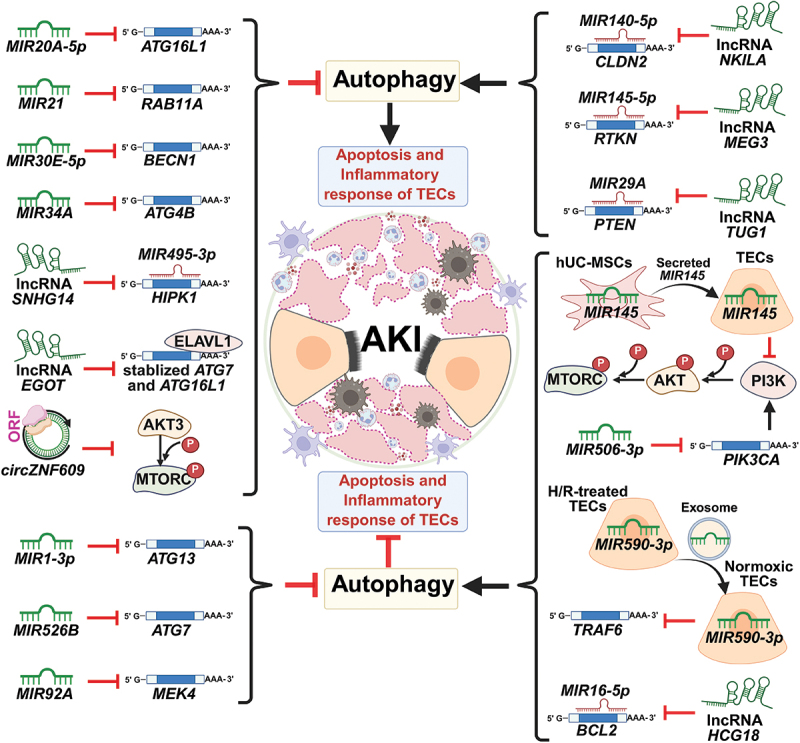
Dual regulation of AKI progression by autophagy-modulating ncRNAs. ncRNAs demonstrate complex, context-dependent regulation of autophagy in AKI, exhibiting both protective and detrimental effects through distinct mechanisms. On one hand, several ncRNAs exacerbate AKI by suppressing autophagy: (1) miRNAs (*MIR20A-5p*, *MIR21*, *MIR30E-5p*, and *MIR34A*) directly target the genes encoding core autophagy components including ATG16L1, RAB11A, BECN1, and ATG4B, respectively; (2) lncRNAs *SNHG14* and *EGOT* regulate autophagy through ceRNA and RNA-binding protein mechanisms, with *SNHG14* sponging *MIR495-3p* to maintain HIPK1 expression and *EGOT* binding ELAVL1/HuR to destabilize *ATG7* and *ATG16L1* mRNAs; and (3) *circZNF609*-encoded proteins inhibit autophagy via AKT3-MTORC1 signaling. Paradoxically, other ncRNAs (*MIR1-3p*, *MIR526B*, and *MIR92A*) appear protective by similarly inhibiting autophagy through targeting ATG13, ATG7, and MEK4, respectively, suggesting differential outcomes depending on cellular context. Conversely, certain ncRNAs promote AKI progression through autophagy activation, including lncRNAs *NKILA*, *MEG3*, and *TUG1* which function via *MIR140-5p*-CLDN2, *MIR145-5p*-RTKN, and *MIR29A*-PTEN axis, respectively. Meanwhile, other studies reveal protective roles of autophagy-inducing ncRNAs: (1) human umbilical cord mesenchymal stem cells (hUC-MSCs)-derived *MIR145* suppresses the PI3K-AKT-MTORC1 pathway in tubular epithelial cells (TECs); (2) hypoxia/reoxygenation (H/R)-treated TECs-derived exosomes downregulate TRAF6 in recipient normoxic cells; (3) *MIR506-3p* directly targets PIK3CA to inhibit PI3K-AKT-MTORC1 signaling; and (4) lncRNA *HCG18* functions as a ceRNA for *MIR16-5p*, thereby upregulating BCL2 and promoting autophagy. These findings collectively highlight the intricate, cell-type specific regulation of autophagy by ncRNAs in AKI pathogenesis (created with BioRender.com).

The autophagy-related ncRNAs involved in AKI, along with their targets, are provided in Figure 4 and Table 2.

**Table 2.** Non-coding RNAs regulate autophagy in acute kidney injury (AKI).

ncRNAs	Expression in AKI/non-AKI tissue	Animal/human (numbers of clinical samples)	Cell line	Potential target (Direct or Indirect*)	Gain- or loss- of- function studies	Autophagy modulation	Influence on AKI pathogenesis	Model/Treatment	Ref.
*MIR1-3p*	Up	Rat	Rat renal proximal tubular epithelial cell line (NRK-52E)	*ATG13* (Direct)	+/+	Inhibition	Suppression (Apoptosis↓, Fibrosis↓)	Animal: Iodixanol; Cell: Iodixanol	[119]
*MIR145*	–	Human	Human renal proximal tubular cell line (HK-2)	PI3K-AKT-MTOR (Indirect)	+/+	Promotion	Suppression	Animal: -; Cell: AOPP	[125]
*MIR20-5p*	Down	Mouse and human	Human renal proximal tubular cell line (HK-2)	*ATG16L1* (Direct)	+/+	Inhibition	Enhancement	Animal: I/R; Cell: H/R	[115]
*MIR21*	Up	Rat	Rat renal proximal tubular epithelial cell line (NRK-52E)	*RAB11A* (Direct)	+/+	Inhibition	Enhancement (Apoptosis↑)	Animal: I/R; Cell: H/R	[114]
*MIR30B*	Up	Human	Human renal proximal tubular cell line (HK-2)	–	+/+	Inhibition	Enhancement (Apoptosis↑, Inflammation↑)	Animal: -; Cell: LPS	[116]
*MIR30E-5p*	Up	Human	Human renal proximal tubular cell line (HK-2)	*BECN1* (Direct)	+/+	Inhibition	Enhancement (Apoptosis↑)	Animal: -; Cell: Urografin	[118]
*MIR34A*	Up	Mouse	Primary mouse proximal tubular epithelial cells	*ATG4B* (Direct)	+/+	Inhibition	Enhancement	Animal: I/R; Cell: -	[117]
*MIR506-3p*	–	Mouse	Mouse renal tubular epithelial cells	*PIK3CA* (Direct)	+/+	Promotion	Suppression (Apoptosis↓)	Animal: LPS; Cell: LPS	[123]
*MIR526B*	Down	Mouse and human	Human renal proximal tubular cell line (HK-2)	*ATG7* (Direct)	+/+	Inhibition	Suppression (Cell viability↑)	Animal: CLP; Cell: LPS	[120]
*MIR590-3p*	Up	Human; 25 patients consisted of 12 young and 13 elderly AKI patients	Human renal proximal tubular cell line (HK-2)	*TRAF6* (Direct)	+/+	Promotion	Suppression	Animal: -; Cell: H/R	[122]
*MIR92A*	Down	Mouse and human	Human renal proximal tubular cell line (HK-2)	*MEK4* (Direct)	+/+	Inhibition	Suppression (Apoptosis↓)	Animal: I/R; Cell: H/R	[121]
lncRNA *EGOT*	Down	Human	Human renal proximal tubular cell line (HK-2)	ELAVL1/HuR (Direct)	+/+	Inhibition	Enhancement	Animal: -; Cell: H/R	[126]
lncRNA *HCG18*	Down	Mouse and human	Human renal proximal tubular cell line (HK-2)	*MIR16-5p* (Direct)	+/+	Promotion	Suppression (Apoptosis↓, Inflammation↓)	Animal: I/R; Cell: H/R	[129]
lncRNA *MEG3*	Up	Mouse and human	Human renal proximal tubular cell line (HK-2)	*MIR145-5p* (Direct)	-/+	Promotion	Enhancement (Apoptosis↑)	Animal: I/R; Cell: H/R	[132]
lncRNA *NKILA*	Up	Human	Human renal proximal tubular cell line (HK-2)	*MIR140-5p* (Direct)	-/+	Promotion	Enhancement (Apoptosis↑, Inflammation↑)	Animal: - Cell: LPS	[131]
lncRNA *SNHG14*	Up	Human; 20 blood samples from patients with sepsis-induced AKI or healthy controls	Human renal proximal tubular cell line (HK-2)	*MIR495-3p* (Direct)	+/+	Inhibition	Enhancement (Apoptosis↑, Inflammation↑)	Animal: -; Cell: LPS	[128]
lncRNA *TUG1*	Up	Rat and human	Human renal proximal tubular cell line (HK-2)	*MIR29A* (Direct)	±	Promotion	Enhancement (Apoptosis↑, Inflammation↑)	Animal: I/R; Cell: H/R	[130]
*circZNF609*	Up	Rat and human; 119 urine samples from patients with acute heart failure	Human renal proximal tubular cell line (HK-2)	AKT3-MTOR (Indirect)	+/+	Inhibition	Enhancement (Apoptosis↑)	Animal: I/R; Cell: H/R	[127]

*Direct: interactions validated by luciferase reporter assays, RNA immunoprecipitation assays, or RNA affinity-isolation assays. Indirect: expression correlations only.

## Regulation of autophagy in CKD by ncRNAs

Similar to AKI, autophagy is frequently regarded as a protective mechanism in CKD. The progression of CKD may include abnormal autophagy mediated by ncRNAs.

### Regulation of autophagy in CKD by miRNAs

#### Inhibition of autophagy in CKD by miRNAs

*MIR214* suppressed autophagy by inhibiting ULK1 expression, which aggravated kidney enlargement and albuminuria in diabetic kidney cells and tissues [133]. Similarly, *MIR22* inhibited autophagy by binding to *PTEN* mRNA; reducing the expression of LC3-II/I; and increasing the expression of COL4A (collagen type IV alpha), ACTA2 (actin alpha 2, smooth muscle) and SQSTM1/p62 [134]. Additionally, *MIR543* suppresses autophagy and promotes fibrosis by reducing TSPAN8 (tetraspanin 8) expression in HG-induced HK2 cells [135]. Furthermore, *MIR379* binds to *FIS1* (fission, mitochondrial 1) mRNA to inhibit mitophagy. This results in the accumulation of the extracellular matrix, fibrosis, damage to and loss of podocytes, thickening of the glomerular basement membrane, and enlargement of glomeruli in DKD mice [136]. Additionally, *MIR122-5p* prevented autophagy and elevated TGFB1 (transforming growth factor beta 1), COL1A (collagen type I alpha), COL3A (collagen type III alpha), FN1 (fibronectin 1), IL6 (interleukin 6), and IL18 (interleukin 18) expression. It further improved the rate of TUNEL-positive staining in spontaneous hypertensive rat kidneys by reducing FOXO3 (forkhead box O3) expression [137]. *MIR376B* inhibits ATG5 expression and promotes collagen buildup and interstitial fibrosis in the kidneys of mice with CKD induced by adenine gavage and a phosphate-rich diet [138]. *MIR34A* inhibits ATG4B expression, elevating ROS levels and inflammation in human renal glomerular endothelial cells induced by HG [139].

#### Promotion of autophagy in CKD by miRNAs

Exosomal *MIR125B* from mesenchymal stem cells boosts autophagy and prevents cell death by binding to the 3' UTR of *TRAF6* mRNA, thus lowering its expression in HG-treated HKCs [140]. The exosomal *MIR25-3p*, originating from M2 macrophages, suppresses DUSP1 (dual specificity phosphatase 1) expression by binding the 3' UTR of its mRNA, consequently fostering autophagy in podocytes and mitigating the damage caused by HG levels [141]. Similarly, *MIR29A* bound to the 3' UTR sequence of *HMOX1* mRNA triggers autophagy and reduces cell death in HG-induced podocytes [142]. Furthermore, *MIR4516* improves mitochondrial autophagy and boosts mitochondrial activity by suppressing SIAH3 (siah E3 ubiquitin protein ligase family member 3), an E3 ubiquitin ligase that reduces PINK1 buildup in impaired mitochondria. This ameliorated renal cell death and fibrosis in mice [143].

### Regulation of autophagy in CKD by lncRNAs

#### Inhibition of autophagy in CKD by lncRNAs

Aberrant expression of lncRNAs enhances CKD progression by inhibiting autophagy. SIRT1 triggers autophagy through the deacetylation of autophagy-associated genes, such as *BECN1*. lncRNA *RISA* suppresses SIRT1 phosphorylation at Ser27 in mice with DKD and those receiving HG treatment of mesangial progenitor cells, thereby diminishing its functionality and expression. This reduces GSK3B (glycogen synthase kinase 3 beta) phosphorylation at Ser9, consequently worsening autophagy, podocyte impairment, proteinuria, and glomerular damage in DKD mice [144]. lncRNA *H19* aggravates puromycin-induced podocyte damage by inhibiting lncRNA *H19-*DIRAS3 (DIRA family GTPase 3)-mediated autophagy [145]. In addition to nonselective autophagy, lncRNAs mediate disease progression by regulating selective autophagy in DKD. For example, lncRNA *SNHG17* impairs PRKN-dependent mitophagy by inhibiting STK4/MST1 (serine/threonine kinase 4) degradation, thereby promoting podocyte apoptosis [146]. In addition, lncRNA *LINC279227* silencing improved downregulation of the expression of mitophagy-related proteins PINK1 and PRKN. However, it decreased the expression of phosphorylated dynamin-related protein and increased the expression of MFN2 (mitofusin 2), which ameliorates the inhibition of mitophagy in HG-treated RTEC and alleviates mitochondrial fusion/fission perturbation [147].

#### Promotion of autophagy in CKD by lncRNAs

lncRNA *HOXB3OS* enhances autophagy in HG-exposed MPC5 cells by disrupting the AKT-MTOR signaling pathway, possibly due to an increase in SIRT1 expression levels [148]. lncRNA *AA465934* binds to *ZFP36/TTP* (ZFP36 ring finger protein), which inhibits the TTP-PIM2 (Pim-2 proto-oncogene, serine/threonine kinase) interaction. This action reduces HMGB1 (high mobility group box 1) levels, reverses the downregulation of autophagy, and alleviates podocyte injury and diabetic nephropathy caused by HG [149].

### Regulation of autophagy in CKD by ceRNA-miRNA axes

#### Inhibition of autophagy in CKD by ceRNA-miRNA axes

lncRNA *SPAG5-AS1* suppresses autophagy through two complementary mechanisms by stabilizing *SPAG5* mRNA through *MIR789-5p* sequestration, while simultaneously inhibiting USP14-mediated SPAG5 protein degradation. Consequently, this dual action activates the AKT-MTOR pathway, ultimately exacerbating podocyte apoptosis [147].

#### Promotion of autophagy in CKD by ceRNA-miRNA axes

lncRNA *XIST* (X inactive specific transcript) targets *MIR30D-5p*, regulates BECN1 expression, ameliorates HG-induced autophagy decline in MPC5 cells, and inhibits cell apoptosis [150]. Similarly, lncRNA *SOX2-OT* acts as a ceRNA that promotes SIRT1 expression by sponging *MIR9*. This process induces autophagy and alleviates HPC damage caused by HG [151]. circRNAs are recently gaining attention for their role in autophagy regulation during the progression of CKD. As revealed by transcriptome sequencing, decrease in *circ0000953* expression was found in podocytes from STZ-induced diabetic mice. Also, the deficiency of a homologous sequence of *circ0000953* was found in kidney biopsy samples from DKD patients. *circ0000953* directly binds to *MIR665-3p* to regulate autophagy process in an ATG4B-dependent manner, suggesting it could be a potential therapeutic target for prevention and treating DKD [152].

**Figure 5.**
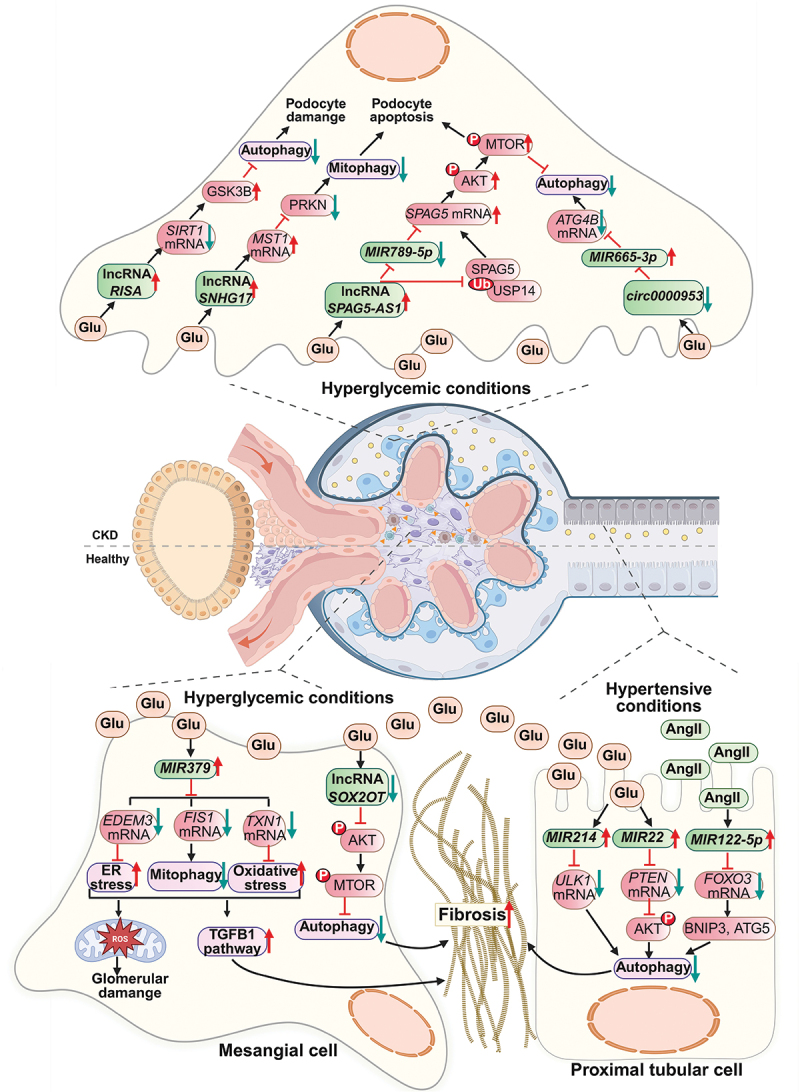
Dysregulation of ncRNAs in renal cells impairs autophagy and promotes fibrosis in CKD. Under diabetic hyperglycemic conditions, podocytes experience dysregulation of multiple ncRNAs that collectively impair autophagy and mitophagy, aggravating cellular injury. *SNHG17* disrupts PRKN-mediated mitophagy by blocking MST1 degradation, thereby promoting podocyte apoptosis. *SPAG5-AS1* inhibits autophagy via dual mechanisms: stabilizing *SPAG5* mRNA by sequestering *MIR789-5p* and preventing USP14-mediated SPAG5 degradation, ultimately activating the AKT-MTOR pathway. Concurrently, elevated *RISA* enhances GSK3B expression in a SIRT1-dependent manner, further suppressing autophagy, while downregulated *circ0000953* fails to sequester *MIR665-3p*, leading to reduced ATG4B and impaired autophagic flux. Together, these disruptions drive podocyte dysfunction in diabetic kidney disease. In hyperglycemic mesangial cells, *MIR379* induces renal fibrosis by targeting *EDEM3*, *FIS1*, and *TXN1*, triggering ER stress, oxidative stress, and mitophagy impairment, all of which activate the TGFB1 pathway. Similarly, reduced *SOX2-OT* expression exacerbates fibrotic response by hyperactivating the AKT-MTOR axis, further suppressing autophagy in a synergistic manner. In tubular epithelial cells under hyperglycemia, upregulated *MIR214* and *MIR22* suppress autophagy by inhibiting ULK1 and the PTEN-AKT axis, respectively, promoting renal fibrosis. Similarly, in hypertensive settings, *MIR122-5p* overexpression impairs autophagy by targeting FOXO3-mediated BNIP3 and ATG5 expression, further exacerbating fibrotic progression. These findings underscore the context-specific regulatory roles of miRNAs in autophagy dysregulation during CKD progression (created with BioRender.com).

The autophagy-related ncRNAs involved in CKD and their targets are provided in Figure 5 and Table 3.

**Table 3.** Non-coding RNAs regulate autophagy in chronic kidney disease (CKD).

ncRNAs	Expression in CKD/non-CKD tissue	Animal/human (numbers of clinical samples)	Cell line	Potential target (Direct or Indirect*)	Gain- or loss- of- function studies	Autophagy modulation	Influence on CKD pathogenesis	Model/Treatment	Ref.
*MIR122-5p*	Up	Rat and mouse	Primary mouse renal tubular interstitial fibroblasts	*FOXO3* (Indirect)	+/+	Inhibition	Enhancement (Fibrosis↑, Apoptosis↑)	Animal: Spontaneously hypertensive rats; Cell: Ang II	[137]
*MIR125B*	Down	Mouse and human; 15 pairs of DKD tissues and adjacent normal tissues	Human embryonic kidney epithelial cells	*TRAF6* (Direct)	+/+	Promotion	Suppression (Apoptosis↓)	Animal: -; Cell: HG	[140]
*MIR155-5p*	Up	Rat and human	Rat renal proximal tubular epithelial cell line (NRK-52E)	*PTEN* (Direct)	+/+	Inhibition	Enhancement (Fibrosis↑)	Animal: -; Cell: HG	[153]
*MIR21*	Up	Mouse and human	Primary human podocytes	*FOXO1* (Direct)	+/+	Inhibition	Enhancement (Apoptosis↑)	Animal: KK Ay mice; Cell: HG	[154]
*MIR214*	Up	Mouse and human; renal biopsies from 20 diabetic patients and 14 non diabetic patients	Renal proximal tubular cell lines (RPTC, BUMPT)	*ULK1* (Direct)	+/+	Inhibition	Enhancement (Fibrosis↑)	Animal: Akita mice, STZ; Cell: HG	[133]
*MIR22*	Up	Rat	Rat renal proximal tubular epithelial cell line (NRK-52E)	*PTEN* (Direct)	+/+	Inhibition	Enhancement (Fibrosis↑)	Animal: STZ; Cell: HG	[134]
*MIR25-3p*	Down	Mouse	Mouse podocytes	*DUSP1* (Direct)	-/+	Promotion	Suppression (Fibrosis↓, Apoptosis↓)	Animal: -; Cell: HG	[141]
*MIR29A*	Down	Mouse	Mouse podocytes	*HMOX1* (Direct)	±	Promotion	Suppression (Apoptosis↓)	Animal: -; Cell: HG	[142]
*MIR34A*	Up	Mouse and human; DKD patient and MCD patient tissues	Human podocytes	*SIRT1* (Indirect)	+/+	Inhibition	Enhancement (Apoptosis↑)	Animal: STZ; Cell: AGEs	[155]
*MIR376B*	Up	Mouse	Primary mouse kidney fibroblasts	*ATG5* (Direct)	+/+	Inhibition	Enhancement (Fibrosis↑, Apoptosis↑)	Animal: High-Phosphate Diet Model;Cell: -	[138]
*MIR379*	Up	Mouse	Primary mouse mesangial cells	*FIS1* (Direct)	+/+	Inhibition	Enhancement (Fibrosis↑)	Animal: STZ; Cell: HG	[136]
*MIR4516*	Down	Mouse and human	Human proximal tubular epithelial cells (TH1)	*SIAH3* (Indirect)	-/+	Promotion	Suppression (Fibrosis↓)	Animal: Adenine Diet Model; Cell: p-Cresol	[143]
*MIR543*	Down	Mouse and human	Human renal proximal tubular cell line (HK-2)	*TSPAN8* (Direct)	+/+	Inhibition	Enhancement (Fibrosis↑)	Animal: db/db; Cell: HG	[135]
lncRNA *AA465934*	Down	Mouse	Mouse podocytes cell line (MPC5)	*TTP* (Direct)	±	Promotion	Suppression (Apoptosis↓)	Animal: db/db; Cell: HG	[149]
lncRNA *H19*	Up	Rat	Primary rat podocytes	*DIRAS3* (Indirect)	-/+	Inhibition	Enhancement (Podocytes injury↑)	Animal: Puromycin; Cell: Puromycin	[145]
lncRNA *HOXB3OS*	Down	Mouse	Mouse podocytes cell line (MPC5)	*SIRT1* (Indirect)	±	Promotion	Suppression (Apoptosis↓)	Animal: db/db; Cell: HG	[148]
lncRNA *GM15645*	Down	Mouse	Primary mouse podocytes	–	+/+	Inhibition	Enhancement (Apoptosis↑)	Animal: db/db Cell: HG	[156]
lncRNA *GM5524*	Up	Mouse	Primary mouse podocytes	–	+/+	Promotion	Suppression (Apoptosis↓)	Animal: db/db; Cell: HG	[156]
lncRNA *LINC279227*	Up	Mouse	Mouse renal tubular epithelial cells	–	-/+	Inhibition	Enhancement (mitochondrial dysfunction↑)	Animal: db/db; Cell: HG	[147]
lncRNA *RISA*	Up	Mouse	Mouse podocytes cell line (MPC5)	*SIRT1* (Indirect)	+/+	Inhibition	Enhancement (Podocytes injury↑)	Animal: db/db; Cell: HG	[144]
lncRNA *SNHG17*	Up	Mouse	Mouse podocytes cell line (MPC5)	*MST1* (Direct)	+/+	Inhibition	Enhancement (Apoptosis↑)	Animal: STZ; Cell: HG	[146]
lncRNA *SOX2-OT*	Down	Human	Human podocytes	*MIR9* (Direct)	+/+	Promotion	Suppression (Apoptosis↓)	Animal: -; Cell: HG	[151]
	Down	Mouse	Mouse mesangial cells	*AKT-MTOR* (Indirect)	±	Promotion	Suppression (Fibrosis↓)	Animal: STZ; Cell: HG	[157]
lncRNA *SPAG5-AS1*	Up	Human	Human podocytes	*MIR769-5p* (Direct)	+/+	Inhibition	Enhancement (Apoptosis↑)	Animal: -; Cell: HG	[158]
lncRNA *XIST*	Down	Mouse	Mouse podocytes cell line (MPC5)	*MIR30D-5p* (Direct)	±	Promotion	Suppression (Fibrosis↓, Apoptosis↓)	Animal: STZ; Cell: HG	[150]
*circ0000953*	Down	Mouse and human; renal biopsies from 8 T1D patients and 13 T2D patients	Mouse podocytes cell line (MPC5)	*MIR665-3p* (Direct)	+/+	Promotion	Suppression (Podocytes injury↓, Inflammation↓)	Animal: STZ; Cell: HG	[152]

*Direct: interactions validated by luciferase reporter assays, RNA immunoprecipitation assays, or RNA affinity-isolation assays. Indirect: expression correlations only.

## ncRNAs regulate autophagy in other kidney diseases

### Kidney stone

The serum and renal tissue mRNA levels in 20 patients with kidney stones revealed significantly increased *MIR155* expression. Similarly, *MIR155* expression was upregulated in a renal tubular cell model of kidney stones constructed using CaOx crystal treatment. Upregulation of *MIR155* expression activates autophagy and promotes cell damage by inhibiting the expression of its downstream target, PIK3CA. This subsequently inhibits the PI3K-AKT-MTOR signaling pathway. Contrastingly, *MIR155* silencing or treatment with the autophagy inhibitor 3-MA ameliorated the cellular damage induced by CaOx crystals [159]. Furthermore, reduced *MIR20B-3p* expression levels have been observed in the urine of patients with hyperoxaluria and in rat models of ethylene glycol-induced hyperoxaluria. Treatment with *MIR20B-3p* extracted from fat-derived matrix cells inhibited oxalate-induced cellular autophagy and inflammation by targeting the downstream molecule ATG7 and reducing its expression [160].

### IgA nephropathy

In mesangial cell-derived exosomes treated with aIgA1, *MIR4455* expression is significantly upregulated and can be transferred by exosomes from mesangial cells to podocytes. Exosomal *MIR4455* directly targets ULK2 and downregulates its expression. This suppresses autophagy and induces podocyte injury [161].

### Lupus nephritis

lncRNA *HOXA11-OS* expression was elevated in lupus nephritis tissues, serum, and cells, whereas *MIR124-3p* expression was decreased. lncRNA *HOXA11-OS* acts as a competitive endogenous RNA for *MIR124-3p* to CCN1/CYR61 (cellular communication network factor 1) expression. This consequently enhances lupus nephritis-IgG-induced autophagy and aggravates podocyte injury. However, sh-*HOXA11-OS* adeno-associated virus significantly attenuated kidney injury in mice with lupus [162].

### Focal and segmental glomerulosclerosis (FSGS)

In a rat model of FSGS induced by 5/6 nephrectomy, glomerular expression of autophagy-related proteins (LC3B, BECN1, ATG7, and ATG5) was significantly downregulated, concomitant with glomerular injury. miRNA profiling revealed upregulated levels of *MIR34C*, *MIR132*, and *MIR214* in the renal tissue of these FSGS rats. Functional studies showed that transfection with *MIR34C*, *MIR132*, or *MIR214* mimics suppressed key autophagy-related protein expression in podocytes, while their respective antagonists restored it. These results imply that *MIR34C*, *MIR132*, and *MIR214* promote FSGS progression by impairing autophagy, suggesting their therapeutic targeting as a potential strategy [163].

The autophagy-related ncRNAs involved in other kidney diseases and their targets are provided in Table 4.

**Table 4.** Non-coding RNAs regulate autophagy in other kidney diseases.

ncRNAs	Disease	Expression in disease/non-disease tissue	Animal/human (numbers of clinical samples)	Cell line	Potential target (Direct or Indirect*)	Gain- or loss- of- function studies	Autophagy modulation	Influence on disease pathogenesis	Model/Treatment	Ref.
*MIR155*	Kidney stone	Up	Human; peripheral blood samples from 20 patients with calcium oxalate nephrolithiasis and 20 normal volunteers	Human renal proximal tubular cell line (HK-2)	*PIK3CA* (Direct), *RHEB* (Direct)	+/+	Promotion	Enhancement (Apoptosis↑, Inflammation↑)	Animal: -; Cell: CaOx crystal	[159]
*MIR20B-3p*	Kidney stone	Down	Rat and human; urine samples from 30 kidney stone patients and 30 healthy controls	Rat renal proximal tubular epithelial cell line (NRK-52E)	*ATG7* (Direct), *TLR4* (Direct)	±	Inhibition	Suppression (Inflammation)↓	Animal: Ethylene glycol; Cell: Oxalate	[160]
*MIR4455*	IgA nephropathy	Up	Human	Primary human podocytes; human glomerular mesangial cells	*ULK2* (Direct)	+/+	Inhibition	Enhancement (Apoptosis↑)	Animal: -; Cell: aIgA1	[161]
*MIR214, MIR132, MIR34C*	Focal segmental glomerulos-clerosis	Up	Rat	Primary rat podocytes	*BECN1*, *LC3B*, *ATG7*, *ATG5* (Indirect)	+/+	Inhibition	Enhancement	Animal: 5/6 nephrectomy models, adriamycin; Cell: -	[163]
lncRNA *HOXA11-OS*	Lupus nephritis	Up	Mouse and human; Serum of 20 patients with diagnosed LN and 10 healthy people	Mouse podocytes cell line (MPC5)	*MIR124-3p* (Direct)	+/+	Promotion	Enhancement (Podocytes injury↑)	Animal: MRL/lpr female mice; Cell: Serum IgG from patients with lupus	[162]

*Direct: interactions validated by luciferase reporter assays, RNA immunoprecipitation assays, or RNA affinity-isolation assays. Indirect: expression correlations only.

## Clinical applications of non-coding RNAs by autophagy regulation in kidney diseases

ncRNAs are crucial in the development of kidney diseases and are increasingly recognized as both diagnostic biomarkers and therapeutic targets, particularly in relation to autophagy regulation. This section systematically discusses diagnostic biomarkers, therapeutic targets, emerging technologies, and existing challenges in the field. In addition, an overview of clinical applications of non-coding RNAs in kidney diseases along with the pros/cons-limitations is provided in Table 5.

**Table 5.** Overview of clinical applications of non-coding RNAs.

Applications	ncRNA	Advantages	Limitations	Example in Kidney Diseases [Ref.]
Diagnostics	miRNA	Small size, highly conserved sequences, high detectability; body fluid stability, noninvasive diagnostic compatibility	High sequence homology (difficult differentiation); individual miRNA specificity limitations	Elevated urinary *MIR452* enables early detection of sepsis-AKI (AUC = 0.90, sensitivity 87.2%), showing strong correlation with creatinine (*r* = 0.83) [164]
	lncRNA	Strong tissue-specific expression, potentially higher disease specificity	Complex structures, low stability; low abundance requires high detection sensitivity	Combined detection of conservative lncRNAs (*HILPDA* and *PRND*) in blood enable early PC-AKI diagnosis (AUC 0.885/0.875 respectively) with 100% sensitivity and 83.93% specificity [165]
	circRNA	Covalently closed circular structure (exonuclease-resistant, highly stable); enhanced detection sensitivity in fluids	Circularity complicates amplification/sequencing (requires specialized primers/algorithms); underdeveloped databases and standardized assays	*circ0072463* (AUC = 0.866) serves as a diagnostic biomarker for septic AKI (78.8% sensitivity, 87.9% specificity), with plasma levels positively correlating with creatinine (*r* = 0.725) [166]
Therapeutic	miRNA	Rapid gene network modulation (mimics/antagomirs); clinical trials underway	Off-target effects (single miRNA regulates hundreds of genes); delivery challenges (e.g., poor in vivo stability)	The oligonucleotide therapy RGLS4326 (phase I trial) treats ADPKD by inhibiting *MIR17* [167]
	lncRNA	lncRNAs often exhibit cell- or tissue-specific expression (e.g., *H19* in placental and tumor tissues) and are closely associated with particular diseases (e.g., cancer), reducing off-target effects	Large molecular size complicates delivery; lncRNAs function via secondary/tertiary structures, yet small-molecule drugs targeting these configurations remain technically immature; low human-mouse lncRNA conservation poses challenges for preclinical studies	Exosome-transmitted lncRNA *ARSR* confers sunitinib resistance in renal cancer by acting as a ceRNA (sponging *MIR34* or *MIR449*) to activate AXL-STAT3 signaling, representing a therapeutic target for resistance reversal [168]
	circRNA	Acts as miRNA sponges, regulates transcription, or encodes functional peptides, offering diverse therapeutic mechanisms; engineered circRNAs can deliver siRNA, mRNA, or proteins, leveraging their stability for drug delivery	Circularization and large-scale production challenges (low backsplicing efficiency/methods requiring optimization); In vivo delivery hurdles (targeting specificity/immune barriers/cellular uptake limitations)	The *circASAP1-*HNRNPC-GPX4 axis promotes ccRCC progression by regulating ferroptosis, with high *circASAP1* expression indicating poor prognosis and serving as a therapeutic target [169]

### ncRNAs as autophagy-related diagnostic biomarkers

ncRNAs play a significant role in the pathogenesis of various kidney diseases and demonstrate strong potential as diagnostic and prognostic biomarkers [170–172]. Their stability in biological fluids such as serum and urine makes them attractive candidates for noninvasive diagnostic tools [173,174]. Recent studies have identified specific miRNAs and lncRNAs with altered expression patterns in kidney diseases including AKI and CKD [18,175–177], where their dysregulation shows clear correlation with disease severity and progression, highlighting their potential for early detection and disease monitoring [178–181]. Clinical evidence supports the biomarker potential of ncRNAs. For instance, sepsis patients with AKI exhibit significantly higher serum and urinary *MIR452* levels than those without AKI. Although NephroCheck – the only FDA-approved AKI detection test – relies on urinary [TIMP2-IGFBP7], urinary *MIR452* shows higher sensitivity (87.23% vs. 61.54%) albeit slightly lower specificity (78.00% vs. 87.18%) [164]. Similarly, *LINC00632/ciRS-7* has emerged as a promising multi-functional biomarker in RCC, with its significant overexpression in tumor tissues and biofluids enabling noninvasive detection, while elevated levels correlate with advanced tumor grade, metastatic potential and poorer overall survival [182]. The dysregulation of ncRNAs has been linked to key pathophysiological processes, including inflammatory and fibrotic responses, often involving abnormal regulation of autophagy [9,183–185]. For instance, *MIR214* and *circ0000953* have been shown to modulate autophagy-related genes and mediate autophagic flux in renal cells, with both being dysregulated in patient renal biopsy samples [133,152].

### Therapeutic targeting of autophagy via ncRNAs

Autophagy induction by drugs, such as rapamycin, or inhibition using chloroquine in mice is prevalent in kidney disease-associated research. Translating these findings into clinical trials is challenging due to the common role of autophagy in maintaining the survival and division of various cells. Additionally, autophagy can cause off-target and unintended side effects in other organs [112,114]. Therefore, leveraging the tissue and cell specificities of ncRNAs for precision therapy could reduce these side effects. The clinical application of ncRNAs as therapeutic targets, particularly in regulating autophagy, is a rapidly evolving and promising area of biomedical research.

Several miRNAs regulate autophagy either by directly targeting autophagy-related genes or by modulating signaling pathways that affect autophagic flux. For instance, treatment with curcumin upregulates *MIR148* and ADAMTS18 (ADAM metallopeptidase with thrombospondin type 1 motif 18) expression in 786-O cells while decreasing the protein levels of LC3-II:LC3-I. This orchestrated response inhibits autophagy, thereby curbing RCC proliferation [113]. Dihydromyricetin improves autophagy and attenuates renal interstitial fibrosis by modulating the *MIR155-5p-*PTEN and PI3K-AKT-MTOR signaling pathways [186]. In vitro, pyridoxamine inhibits advanced glycation end products (AGEs) formation, which in turn prevents the expression and activation of TP53-*MIR34A* induced by AGEs. This consequently restores SIRT1 expression to mitigate podocyte apoptosis [155]. Atrasentan alleviates kidney injury by inhibiting *MIR21* expression and fostering cellular autophagy in mice with diabetic kidney disease [154]. In addition, some lncRNAs and circRNAs play similar roles. For example, the total glucosides of paeony can reduce lncRNA *HCG18* expression level to facilitate cellular autophagy and suppress apoptosis in IRI-induced AKI [129]. Furthermore, Shensu IV promotes glomerular podocyte autophagy by modulating the expression of lncRNA *H19-*DIRAS3, thereby exerting protective effects on podocytes [145].

miRNA mimics and inhibitors are primary tools for modulating miRNA expression in therapeutic applications. For instance, *MIR365-3p* mimics decrease target gene *Rheb* expression, activate autophagy, and mitigate cell apoptosis induced by CM in NRK-52E cells [124]. Moreover, *MIR92A* agomir significantly reduces the apoptosis and autophagy in HK2 cells, induced by hypoxia, hypoxia-reoxygenation, and rapamycin, while *MIR92A* antagomir has opposite effects [121]. Locked Nucleic Acid (LNA)-anti-*MIR214* acts as an inhibitor of *MIR214*, preventing the reduction of ULK1 expression and autophagy damage in diabetic kidneys, thereby reducing kidney hypertrophy and albuminuria [133].

### Emerging technologies in ncRNAs-based diagnostics and therapeutics

Emerging technologies, including nanoparticles, clustered regularly interspaced short palindromic repeats (CRISPR), and single-cell sequencing, show promising potential for diagnostics and therapeutics related to ncRNAs.

Nanomedicine-based therapies provide significant advantages in drug delivery by improving drug solubility and reducing off-target effects. An expanding range of engineered nanomaterials shows significant potential for renal disease applications, leveraging their distinctive physicochemical properties to achieve targeted kidney delivery and enhanced therapeutic efficacy [187]. Nanoparticles can either be combined with miRNA inhibitors or drugs to decrease miRNA expression. For example, combining anti-miRNA plasmids with iron oxide/alginate nanoparticles linked to anti-kidney antibodies allows for the targeted delivery to renal tubular cells. The anti-miRNA plasmids released from these nanocomposites effectively inhibited cell proliferation and cyst formation in both cellular and animal models of PKD [188]. Moreover, gold nanoparticles (AuNPs) conjugated with dapagliflozin inhibit apoptosis and fibrosis in DKD through their suppressive effects on *MIR192* and *MIR21* [189]. Conversely, nanoparticles can also deliver miRNA mimics or drugs to enhance miRNA expression. For example, polyethylenimine nanoparticles (PEI-NPs) carrying *MIR146A* significantly increases *MIR146A* expression compared to control groups, while simultaneously reducing renal fibrosis area, decreasing ACTA2 expression, and limiting infiltration of ADGRE1/F4/80^+^ macrophages into the obstructed kidney [190]. Furthermore, dioscin-loaded zein nanoparticles were demonstrated to alleviate LPS-induced acute kidney injury by modulating the MIRLET7I-TLR4 (toll like receptor 4) signaling pathway [191].

CRISPR and its associated Cas enzymes have recently become powerful diagnostic tools [192]. Stevens et al. built on this technology and integrated nanomedicine approaches to develop an innovative nanozyme-based CRISPR diagnostic platform called CrisprZyme. This platform enables quantitative detection of non-coding RNA species with high sensitivity, without the need for preamplification [193]. In parallel developments, Chen and colleagues successfully employed an RNA-targeting CRISPR-Cas13 system (RfxCas13d) to specifically distinguish circular RNAs from their linear mRNA counterparts by designing guide RNAs (gRNAs) that precisely target back-splicing junction (BSJ) sites. The RfxCas13d-BSJ-gRNA system outperforms conventional shRNA-mediated approaches. It achieves more efficient circRNA knockdown and significantly reduces off-target effects on linear mRNAs [194].

Recent breakthroughs in single-cell RNA sequencing (scRNA-seq) have revolutionized the study of ncRNA dynamics in renal pathophysiology, offering single-cell resolution to dissect disease mechanisms [195]. By delineating cell subtype-specific ncRNA expression profiles within the kidney, this approach enables precise identification of disease-specific therapeutic targets and facilitates the rational design of optimized nanomedicine systems. Furthermore, when integrated with CRISPR-based screening or spatial transcriptomics, scRNA-seq can transform our understanding of how ncRNA-autophagy networks are regulated in different renal compartments. This integration will help advance personalized therapeutic strategies. Huang et al. made a significant breakthrough by developing SUPeR-seq (single-cell universal poly(A)-independent RNA sequencing), an innovative method that utilizes random primers containing fixed anchor sequences instead of traditional oligo(dT) primers for cDNA synthesis. This approach demonstrated remarkable sensitivity for circRNA detection at single-cell resolution, identifying 141 circRNA transcripts in individual HEK293 cells and 2,891 in single mouse early embryonic cells [196].

### Current challenges

The clinical use of ncRNA-based diagnostic biomarkers and therapeutic targets presents several challenges. Regarding diagnostic biomarkers, ncRNAs are still largely understudied in biological fluids that indicate renal cell-specific autophagic activity. There is a lack of noninvasive tools that reflect autophagic activity in injured kidney cells. Therefore, analyzing the expression profiles of tissue and cell type-specific ncRNAs in biological fluids may provide a promising noninvasive indicator of autophagic activity in kidney cells. Second, there is an urgent need for standardized methods to quantify ncRNA expression in biological fluids. Third, ncRNAs that may act as biomarkers need to be validated in large cohorts to establish their reliability and specificity for various kidney diseases.

From the perspective of diagnostic biomarkers: First, bioinformatic analysis shows that miRNA has multiple potential targets. This hinders the accurate identification of its mechanism of action. Additionally, off-target effects may occur since the target sequences of miRNAs are prevalent across many genes and genomic regions [197]. Moreover, the typically low conservation of lncRNAs across species hinders the identification of their specific functions. This prevents the translation of discoveries made in murine models to human applications, such as lncRNA *LFAR1* and lncRNA *TSI* [18,118]. Second, similar to all RNA treatments, another significant challenge for ncRNA therapy is delivery. Effective delivery vectors require the efficient delivery of ncRNA to target cells and avoidance of triggering immune responses or toxic reactions [198,199]. In contrast, circRNAs such as *circBNC2* are highly conserved across species, including in humans and mice [177,200]. Given their stability, tissue specificity, and conservation, circRNAs could be a promising strategy for preventing and precisely treating various diseases when delivered via nanoparticles [201], adeno-associated viruses [202], or exosomes for targeted therapy [203,204]. Third, significant regulatory hurdles must be overcome as ncRNA-based therapies progress toward clinical trials. The long-term safety and efficacy of these therapies must be thoroughly evaluated in clinical settings, which can be complicated by the complexity of ncRNA interactions and their diverse roles in cellular processes [205].

## Conclusions and future perspectives

This review provides a comprehensive exploration of how ncRNAs regulate autophagy in the kidney and thus affect disease progression. First, we reviewed the effects of miRNAs, lncRNAs, circRNAs and ceRNA-miRNA axes on the promotion or inhibition of autophagy in RCC, AKI and CKD, which improved or aggravated the progression of the disease. Second, although there are fewer studies, we have also seen similar effects in kidney stone, IgA nephropathy, lupus nephritis and FSGS. Finally, we illustrate the clinical potential of ncRNAs as biomarkers and therapeutic targets for kidney diseases, while also highlighting the development of emerging technologies – such as nanoparticles, CRISPR, and single-cell sequencing – alongside existing challenges.

As a stress-response mechanism, autophagy is activated under various cellular stress conditions – including ROS, ER stress, and drug-induced stress. These stressors can regulate ncRNAs, which in turn may modulate autophagy and contribute to disease pathogenesis, including kidney disease. Key examples include: During ER stress, *MIR346* induction stimulates autophagic flux in HeLa cells, providing cytoprotection against stress-induced cell death [206]. lncRNA *IGFL2-AS1* is upregulated in sunitinib-resistant RCC cells, where it enhances autophagy by competitively binding HNRNPC to promote TP53INP2 expression, leading to tumor progression [107]. Although systematic classification of stress conditions and related ncRNAs would elucidate molecular pathways in kidney disease, current evidence primarily comes from complex pathological contexts rather than defined stress models. Future research should employ controlled stress paradigms (e.g., tunicamycin for ER stress, H_2_O_2_ for oxidative stress) to precisely map stress-specific ncRNA signatures and their autophagy-modulating functions in renal pathophysiology.

Additionally, selective autophagy, which includes lipophagy, mitophagy, aggrephagy, and reticulophagy, plays a crucial role in kidney disease, but its effects can vary depending on the context. Lipophagy controls the turnover of lipid droplets in kidney tubular cells. When it is impaired in DKD, it worsens lipotoxicity and fibrosis. Studies have linked a deficiency in autophagy to increased lipid accumulation [207]. Mitophagy maintains mitochondrial quality control, mitigating oxidative stress and inflammation; however, dysregulated mitophagy due to sustained ROS overproduction can paradoxically amplify tubular injury in AKI and CKD [208,209]. HIF1A-BNIP3-mediated reticulophagy helps protect against IRI by degrading stressed ER components. However, if this pathway is overactivated, it may lead to the depletion of essential organelles, indicating the need for careful therapeutic targeting [210]. Recent evidence indicates that ncRNAs can adjust these selective autophagy pathways. They may help restore balance or worsen disease, depending on how they interact with autophagy regulators such as ULK1 and OFD1 [211]. Thus, understanding the spatiotemporal regulation of selective autophagy and its crosstalk with ncRNAs is critical for developing context-specific therapies.

However, while we have noted the protective role of autophagy in kidney disease, it is important to understand that it can also have harmful effects. This balance depends on various factors, including the type of injury and the stage of the disease. For example, in various types of AKI, the pharmacological autophagy inhibitor 3-MA has been shown to worsen cisplatin-induced tubular cell apoptosis while improving LPS-induced renal inflammation [84,87]. Furthermore, during the early phase of AKI, autophagy plays a protective role by clearing damaged organelles and mitigating oxidative stress [212]. However, during the transition from AKI to CKD, or in established CKD, prolonged activation of autophagy may promote renal fibrosis via the FGF2-EGR1 pathway [13,14,213].

Intriguingly, autophagy can also target ncRNAs by participating in ncRNAs clearance, but this area of research has received little attention, and existing evidence is sparse. To date, lncRNA *PVT1* is the only lncRNA discovered to be regulated by autophagy. In diabetes, the expression of lncRNA *PVT1* is significantly elevated, but the autophagy inhibitor 3-MA markedly reduces lncRNA *PVT1* levels [214].

Overall, targeting autophagy-related ncRNAs is a novel and innovative approach that could significantly advance the diagnosis and the treatment of kidney diseases. Through continuous research and innovation, we look forward to developing more effective and safer therapies that will bring new hope to patients.
